# Predictive Models of Dairy Cow Thermal State: A Review from a Technological Perspective

**DOI:** 10.3390/vetsci9080416

**Published:** 2022-08-08

**Authors:** Soraia F. Neves, Mónica C. F. Silva, João M. Miranda, George Stilwell, Paulo P. Cortez

**Affiliations:** 1 CEFT—Transport Phenomena Research Centre, Faculty of Engineering, University of Porto, Rua Dr. Roberto Frias, 4200-465 Porto, Portugal; 2 ALiCE—Associate Laboratory in Chemical Engineering, Faculty of Engineering, University of Porto, Rua Dr. Roberto Frias, 4200-465 Porto, Portugal; 3 CIISA—Animal Behaviour and Welfare Laboratory, Associate Laboratory for Animal and Veterinary Sciences (AL4AnimalS), Faculty of Veterinary Medicine, University of Lisbon, 1300-477 Lisbon, Portugal; 4 ICBAS-UP—Institute of Biomedical Sciences Abel Salazar, University of Porto, Rua Jorge Viterbo Ferreira 228, 4050-313 Porto, Portugal; 5 CECA/ICETA—Centre for Animal Science Studies, Rua D. Manuel II, Apartado 55142, 4051-401 Porto, Portugal

**Keywords:** dairy cow, heat stress, cow thermal state, predictive model, bioclimatic indexes, machine learning, mechanistic models, numerical model

## Abstract

**Simple Summary:**

Heat stress in cattle is broadly defined as a physiological condition in which body temperature rises, and the animals are no longer able to adequately dissipate body heat to maintain thermal equilibrium due to environmental factors. Dairy cattle are particularly sensitive to heat stress because of the higher metabolic rate needed for milk production. Due to global warming and the expected growth of milk production in warmer regions, an increase in the occurrence of heat stress can only be avoided with the use of environmental control systems. However, most available systems were developed to take corrective measures or are not accurate enough to effectively prevent heat stress, as there is not yet an automated technological solution that considers all the environmental and animal variables that determine the occurrence of this condition. Further, these systems must be connected in time to prevent this condition in cattle but also disconnected when they are no longer needed, as their use raises major economic and environmental concerns regarding energy and water consumption. This review describes and discusses three types of predictive models that can make these systems more effective in preventing heat stress and more efficient in the use of energy and water.

**Abstract:**

Dairy cattle are particularly sensitive to heat stress due to the higher metabolic rate needed for milk production. In recent decades, global warming and the increase in dairy production in warmer countries have stimulated the development of a wide range of environmental control systems for dairy farms. Despite their proven effectiveness, the associated energy and water consumption can compromise the viability of dairy farms in many regions, due to the cost and scarcity of these resources. To make these systems more efficient, they should be activated in time to prevent thermal stress and switched off when that risk no longer exists, which must consider environmental variables as well as the variables of the animals themselves. Nowadays, there is a wide range of sensors and equipment that support farm routine procedures, and it is possible to measure several variables that, with the aid of algorithms based on predictive models, would allow anticipating animals’ thermal states. This review summarizes three types of approaches as predictive models: bioclimatic indexes, machine learning, and mechanistic models. It also focuses on the application of the current knowledge as algorithms to be used in the management of diverse types of environmental control systems.

## 1. Introduction

Heat stress in cattle is broadly defined as a physiological condition during which the animal is no longer able to regulate its internal temperature within a comfortable level because of an increase in body heat storage [1]. This physiological condition can lead to impaired health and immunity [2,3], deteriorated living conditions and even mortality during extreme events [4,5], especially in younger cattle [6,7]. Due to global warming, the number of heat stress events is expected to increase (2000 h in Central Europe and Mediterranean regions [8]) causing vast economic losses (e.g., the dairy industry loses approximately USD 900 million/year only in the U. S. [3]). Furthermore, the conventional genetic selection of dairy breeds to increase dry matter intake (DMI) and milk yield [9,10] resulted in cows with elevated internal heat loads (due to high milk production) that may lead to heat stress much earlier than their lower-producing counterparts [11,12,13]. For instance, the loss of milk production due to heat stress is expected to increase at a rate of 174 ± 7 kg/cow/decade in the 21st century [14]. Thus, the impact of this syndrome on animals cannot be neglected in any way as the future dairy industry faces the difficult challenge of increasing milk production in warmer environmental conditions [8], while preserving the welfare of dairy animals.

Several strategies are reported in the literature to mitigate heat stress, from cattle housing design to shifting feeding times to cooler periods and shade seeking [3,15,16], but only cooling and forced ventilation systems are effective above certain temperatures and humidity conditions [16]. Further, these systems must be connected in time to prevent the development of heat stress in cattle but also disconnected when they are no longer needed, as their use raises major concerns regarding energy and water consumption, which may be economically and environmentally unsustainable in the near future [17,18,19].

Presently, as a wide range of sensors and equipment have been developed to support farmer routines, it is possible to measure several variables that, with the aid of algorithms based on predictive models, could allow anticipating the thermal state of the animal. This review summarizes three types of approaches with the potential to be used as predictive models: bioclimatic indexes, machine learning, and mechanistic models. The analyses focused on the application of the current knowledge as algorithms to be used in different types of dairy technology (e.g., fans, sprinklers), considering the several input and output variables of the models as practical issues.

## 2. Heat Stressed Dairy Cows and the Available Technological Solutions to Detect and Mitigate Heat Load

### 2.1. Heat Stress Indicators

Heat stress has an enormous impact on animal health, biological functioning, and welfare. One of the most noticeable consequences of heat stress in dairy cows is the reduction of DMI, which causes a drop in milk production by decreasing the availability of nutrients used for milk synthesis [20,21]. Moreover, heat stress compromises cattle welfare by changing or inhibiting social and eating behavior [22], increasing susceptibility to disease [23], and causing stress and discomfort [24]. Under harsh ambient conditions, animals show physiological or behavioral responses or, most often, a combination of both. For instance, in the early stage of heat exposure, the animal body quickly responds to maintain homeostasis. As the amount of heat load increases, the physiological response becomes more evident, triggering an increase in both respiratory (RR; [25,26]) and heart rates (HR; [27,28]). Therefore, cows change their behavior [3,24], e.g., water ingestion, reducing movement and seeking shaded areas to minimize heat load, leading to the subsequent decrease in milk production, and finally a decline in fertility [29]. Although interdependency exists, physiological, behavioral, and production indicators were examined separately to facilitate the analyses [30,31].

#### 2.1.1. Physiological Indicators

The core body temperature in cattle indicates the temperature of the most important organs of the body such as the heart, liver, and brain [32]. It is often used as an indicator of heat stress and typical values of 38.0 to 39.3 °C are observed in non-heat-stressed cattle [27]. Rectal or vaginal temperature is used as a conventional “gold standard” measure of core body temperature [33]. Yan et al. [34] studied the rectal temperature of dairy cows exposed to several heat stress conditions, reporting an increase of 1 °C in rectal temperature (from ~38.5 to ~39.5 °C) from neutral to heat stress conditions. The rectal temperature is the predominant method employed to measure the internal temperature of heat-stressed cows, but other internal regions of the cow body, such as the vagina [35,36], the rumen [16], and the tympanum [37], have also been studied and correlated with typical behavioral and ambient conditions of heat stress. Nordlund et al. [35] monitored the vaginal temperature of 20 high-producing cows, observing a temperature increase during lying bouts and a temperature decrease when the cows were standing in pens (both free stall and milking center holding pens). Another example, reported by Curtis et al. [16], is the delay in the rumen temperature increase of 2 to 5 h when compared to ambient temperature, probably due to the thermal inertia caused by the cow’s substantial body mass. One possible approach to identifying animals affected by heat stress is by monitoring the external temperature of the cow’s body. Several authors have been studying the temperature of eyes, limbs, and udder and have correlated it with core temperature [28,38].

In some situations, the variation of other physiological indicators is more significant than the core body temperature. For example, in moderate heat stress conditions, Vizzoto et al. [27], compared shaded and non-shaded cows at 1300 h (GMT−0200 h) and observed a significant increase in the respiratory and heart rates, but no significant differences in the cow’s body temperature. Cows with access to shade had a lower respiratory rate of 5.9 breaths per minute and a lower heart rate of 20.5 beats per minute when compared with those without access to shade. However, a significant increase in the core temperature of not-shaded cows was observed later at 1700 h. Respiratory rate increase followed by a body temperature increase was also reported by Ferrazza et al. [25] in Holstein cows exposed to intense and prolonged heat stress conditions. Moreover, Brown-Brandl et al. [26] concluded that the respiration rate is a good physiological indicator of heat stress because there was little or no lag associated with it and it was consistently affected in all the categories of the daily maximum temperature–humidity index (THI; i.e., it measures the risk of the animal suffering from heat stress; see Section 3.1).

The endocrine response to heat stress is mainly reflected by elevated glucocorticoids (cortisol), aldosterone, antidiuretic hormone, thyroxine, prolactin, and growth hormone. However, their use as heat stress indicators would imply frequent blood sampling, an invasive procedure that requires the handling of animals. A less invasive measurement is milk cortisol (MC) concentration as it is highly correlated to plasma cortisol concentrations sampled at the same time [39]. Glucocorticoid metabolites (produced by the liver and excreted into the gut via the bile), but not the native glucocorticoids, can be detected in feces [40]. These fecal Cortisol Metabolites (FCM) can thus provide an integrated measure of stress over several hours, whereas MC provides a measure of stress occurring in the previous minutes. Despite being recognized as useful indicators of the occurrence of heat stress in cattle, their use in a heat stress study has several limitations besides laboratory costs. In the case of MC, its measurement as short-term indicators would imply milking cows outside milking hours, which could be a stressful event for the cows.

#### 2.1.2. Behavioural Indicators

Under heat stress conditions, cows tend to adapt their body posture [41,42], spending more time standing to increase the body surface area exposed to air, thus dissipating more heat. Allen et al. [43] showed a direct correlation between the core temperature and the duration of the standing period of lactating dairy cows experiencing mild to moderate heat stress. When a cow’s vaginal temperature exceeded 38.93 °C, there was a 50% likelihood that the cows would be standing. Furthermore, Yan et al. [34] correlated the rectal temperature and temperature–humidity index of cows with different postures (i.e., laying or standing). They observed that recumbent cows showed higher rectal temperature for lower values of THI, which indicates how the laying posture negatively affects the cows’ thermal state.

Heat stress also affects the cow’s disposition to display natural estrous behavior, reducing both the duration and intensity of estrous expression [44,45] and is responsible for a decrease of 20 to 30% in conception rates [46]. Furthermore, the decrease in conception rates during summer is also explained by oocyte quality reduction, early embryonic death, endometrium dysfunction, and reduced spermatogenesis in bulls [47,48].

Although less documented in the literature due to difficult quantification, it is undeniable the important changes in the emotional state, with evident signs of malaise, disorientation and frustration in animals affected by heat stress [3].

Other changes in the social behavior of heat-stressed animals are also reported, such as higher levels of aggression near water drinkers or competition for shade seeking [22,49,50]. Furthermore, the eating and drinking behavior of cows also changes [50]. For example, the increase in heat load is accompanied by an increase in water intake and a significant reduction in dry matter intake (DMI). This reduction in feed intake occurs in all mammalian species under heat stress conditions. However, the extent of this reduction depends not only on environmental conditions but also on the production level [30]. In heat-stressed lactating cows, feed intake may be reduced by as much as 40%, according to the National Research Council of the United States of America [51]. This causes a decline in milk production by decreasing the availability of nutrients used for milk synthesis. Likewise, a change in milk composition is to be expected such as a decline in total protein or fat content [52,53] but also in fat composition [54]. These factors related to the yield and composition of milk are often used as indicators of performance, as described in the following subsection.

#### 2.1.3. Performance-Related Indicators

Under heat stress conditions, the activation of the cow’s thermoregulatory system can increase metabolic maintenance requirements by 7 to 25% [55], exacerbating both the existing metabolic stress and the reduction of milk production [3,30]. However, the drop in milk production has a time delay associated with it, being clearly noticed a few days after the first day of exposure to a high-temperature environment. For example, Van Laer et al. [56] reported a significant decrease in milk yield after a lag period of two days. Nevertheless, as the heat load decreases to thermoneutral conditions, the milk yield progressively returns to values obtained in thermoneutral cows [30].

Another relevant performance-related indicator that might be affected by heat stress is the milk composition which typically presents a reduction of lactose, protein, and/or fat with increasing values of bioclimatic indexes [53,56]. Nonetheless, the decrease in milk production is not always accompanied by a change in the composition of the milk [30].

### 2.2. Methods to Detect Heat-Stressed Cows

The complexity of cow physiological mechanisms and their interdependency with its behavior and with environmental conditions recommend the measuring of indicators at various levels: cow’s body, performance, behavior, and environment. The record of the latter is straightforward, and the parameters often studied are the environment temperature, humidity, air velocity, and solar radiation [57,58]. Several types of equipment are available in the market such as temperature and humidity sensors for indoor/outdoor conditions [23,59], anemometers for measuring wind direction and velocity [8,42,60], and solar radiation sensors [57,61]. However, a broader range of methods may be used to assess animal health and welfare [30], from equipment with different frequencies of data acquisition (e.g., real-time vs. hourly measurements) to continuous or scan observations. Thus, to facilitate the analyses of the methods used to study the animal physiological, behavioral, and performance-related indicators, the methods are described in the following sections by these categories: equipment measurement frequency and observations.

#### 2.2.1. Equipment with Low Data Acquisition Frequency

Over the last decades, manual detection has been the most used method for measuring physiological parameters such as heart rate, sweating rate or rectal temperature [57]. However, these methods can be classified as invasive or, at least, disturbing to the animal, since the interaction with the animal, e.g., by touching or inserting portable equipment in the animal body [33], can add extra stress and consequently influence the measurements. These methods are also time-consuming and may involve significant additional labor costs. The advantage of these type of methods is that it often requires cheap and user-friendly equipment, e.g., a thermometer for rectal temperature measurement [1,25] or a stethoscope for heart rate measurement [25,27].

Sensors coupled with data loggers are also often used to record the measurements over discrete time points [2,57,62]. An evaporimeter was used by Rungruang et al. [63] to measure, four times a day, the sweating rate at the skin or hair-end level. In order to avoid the displacement of cattle from their social group for restrainment in a crush, the surface body temperature is often measured with thermal data loggers, e.g., a neck collar attachment for skin temperature measurement [2].

Another rapidly developing promising alternative is infrared thermography [2]. Thermal imaging cameras for Infrared Thermography (IRT) offer a remote, non-contact method for recording surface temperature and have been explored as a proxy for core body temperature measurements [28]. However, the use of such systems presents important operational challenges since the successful determination of IRT of external body surfaces depends on the effective minimization of confounding factors, e.g., the animal skin and hair, or external factors such as ambient temperature, sunlight or wind [2,64]. In a study that used infrared thermographic images to predict heat stress events in feedlot cattle, Unruh et al. [65] concluded that it was an objective method to monitor beef calves for heat stress in research settings, but at the time, they also noted that thermographic data was of little predictive benefit compared, e.g., with forecasted weather conditions. Like Idris et al. [2], these authors refer that the use of infrared thermography as a diagnostic tool to monitor heat stress in cattle requires further research [65].

The research and development of monitoring technology have been accompanying the modernization of the farm sector in the direction of a more efficient and automatized system (i.e., livestock precision farming). To ensure a prompt response, the new monitoring equipment should provide the continuous monitoring of the process (i.e., herd thermal state and environment conditions), ideally in real-time.

#### 2.2.2. Equipment with High Data Acquisition Frequency

Automated temperature monitoring devices can have several applications in livestock, either to monitor animal health or to support scientists and farmers for precision farming and remote monitoring. In 2018, Koltes et al. [66] analyzed different automated body temperature monitoring technologies and discussed their use to develop new strategies to control potential animal health problems. The authors highlighted that the measurement at the animal level would be useful to manage heat stress and disease, despite the associated investment costs. Furthermore, they pointed out a need for developing software for complex data storage and treatments. A similar conclusion was drawn by Sellier et al. [62]. In order to avoid displacement of cattle from their social group and restraint in a crush, thermal data loggers can be used to measure body temperature by, e.g., a neck collar attachment (for skin temperature), a rumen bolus or a vaginal insert, recording temperature at pre-determined time intervals or having a telemetric option to transmit recorded data in real-time to a user-defined receiver [2]. As an alternative, the core body temperature can be monitored in real-time using implantable biosensors [60].

Rumen boluses provide prompt means of measuring rumen (or reticulum) temperature, but measurements may be influenced by the intake of fluids. One of the challenges in using rumen temperature is the acute impact of water intake [58]. Ingestion of large volumes (10 to 15% rumen volume) of cold water (0 to 8 °C) provokes a sudden drop in rumen temperature (up to 10 °C) within 15 min of water consumption and required approximately 2 to 3 h to return to baseline [67]. However, as water intake is directly related to feed intake, it may also be reduced in heat-stressed animals compared to thermoneutral controls [68]. Both feed and water intake can be monitored through several methods: neck-mounted activity collars, ear tags and/or leg data logger, coupled with micro-electro-mechanical accelerometers. This can provide important data regarding feeding behavior as head movements toward the feed bunk are recorded while a ruminal bolus can provide temperature data [69].

Sensors coupled with data loggers have also been used for high-frequency (minutes) measures that can be considered continuous. Polsky et al. [36] used temperature-recording data loggers coupled to an intra-vaginal progesterone implant for vaginal temperature measurement with a 10 min frequency. Respiration sensors coupled with a micro-computer have also been used to record the respiration rate with high incidence [61,70]. On the downside, battery problems or malfunctions (e.g., sensor displacement or removal) are usually only revealed at the end of the trial.

#### 2.2.3. Observations

Respiration rate and panting serve as early indicators of increasing heat stress [24] and provide an easy method for the non-invasive and distant assessment of heat stress response [48,71], unless cattle are more than approximately 30 m away (at this distance it is difficult to visualize the cow’s behavior [72]). The panting score is often assigned on a scale of 0 to 4, in which zero is no panting and four is severe panting [27,28]. The ethogram for panting scoring can be helpful to assess an individual or a group of animals’ response to heat stress [37,72]. However, human observation adds uncertainty to the collected data and the outcomes are extremely dependent on the researcher or farmer’s experience [2]. Additionally, it entails a substantial rise in labor costs.

The major limitation of these animal-based methods is the moment at which the heat stress is detected. The methods identify the signs of heat stress and not the conditions that potentially lead to heat stress (pre-heat stress conditions), so it fails to prevent the deterioration of the animal’s health.

### 2.3. Cooling Technologies

A wide range of alternative strategies to reduce heat load is used, from shifting feeding times to cooler periods [3] to nutrition adaptation [73]. However, above certain temperature and humidity conditions, only evaporative cooling and forced ventilation systems are effective (e.g., fans [74] and sprinklers [20]). Several environmental control systems for dairy cows exist based on the principles of convection, conduction, radiation, and evaporation and most can mitigate or even avoid heat overload episodes [57,75]. Fan installations, which facilitate air movement and increase convection, can decrease both respiratory rate and rectal temperature and promote DMI [76]. Similar outcomes are achievable with other forms of evaporative cooling systems that make use of high-pressure mist injected into fans or large water droplets from low-pressure sprinkler systems that completely soak the cow’s hair coat [20]. Despite advances in cooling technologies, primary concerns, regarding energy and water consumption, arise with the use of these systems, namely sprinkler systems. Depending on herd size, large volumes of water are needed for cooling, reaching levels of water consumption (215 to 454 L per cow per day) that may become economically and environmentally unsustainable in the near future [17,18]. Furthermore, one should not forget that these systems generate equally large amounts of wastewater that must be managed. Along with drinking water and water needed during the milking routine, water for evaporative cooling is one of the main three uses of potable water in commercial dairies and decreasing water usage and contamination is critical to the sustainability of the industry [19]. Therefore, these cooling systems must be turned on in time to provide immediate thermal relief for dairy cows but also turned off when unnecessary, to avoid wasting energy and water.

## 3. Predictive Models

Determining the pre-heat stress conditions is essential to make environmental control systems more capable of not only effectively preventing heat stress and related conditions, but also making them more efficient in energy and water usage. Taking into account the above-mentioned limitations of the assessment through animals, trying to determine this condition only with environmental variables is the most logical step, as they are more easily measurable using cheaper equipment. To achieve this objective, it will be necessary first to develop algorithms based on predictive models that integrate all data from the environment and animals, between situations transitioning from thermoneutral conditions to thermal stress conditions and vice versa, and combining all variables that affect the cow’s thermal state. This review summarizes three types of approaches with the potential to be used as predictive models: bioclimatic indexes, machine learning, and mechanistic models.

### 3.1. Bioclimatic Indexes

The bioclimatic index is a measure often used to assess the risk of an animal suffering from heat stress. This type of index was developed to model the combined effect of environment, production, and animal welfare indicators, assuming uniform responses from all the animals. As input variables, the indexes consider a combination of environmental conditions, such as temperature, relative humidity, solar radiation, air velocity, and precipitation [30,57,76,77], that are easily monitored (e.g., with weather stations or indoor/outdoor sensors). Typically, these indexes are based on regression equations, usually with three levels of stress (i.e., mild, moderate, and severe) represented by threshold values [57]. Therefore, the implementation of the indexes as an algorithm is straightforward. The main concerns rely either on the selection of the most adequate index or its feasibility to be integrated into a dairy farm cooling system.

The predominant index incorporates only air temperature and relative humidity, i.e., temperature–humidity index (THI; [30,34,78]). Probably due to the minimum requirement of inputs (meteorological parameters) and the consequent simple application, the THI is a popular approach among farmers and veterinary researchers. Several THI indexes have been developed over the years, considering multiple modifications and refinements based on different breeds and using different parameters to categorize heat stress levels (e.g., respiratory rate, rectal temperature). The input and output variables of some bioclimatic indexes are described in Table 1. In fact, the THI is used to assess the thermal comfort of animals, humans, or others. Thom [79] developed a well-known THI index for the evaluation of human discomfort levels in warm weather using two parameters directly related to sensible and latent heat transfer: dry bulb temperature and wet bulb temperature. For livestock, the THI followed the same rationale to study the discomfort of, e.g., bull calves [80], dairy animals (study cited in [81]), or cattle [7], in this case, considering production (e.g., milk and protein yield; [82,83]) and animal comfort factors (painting scores and rectal temperature [7,80]) as indicators of heat stress.

The THI models have been improved by considering more weather parameters and by fine-tuning the thresholds [7,82,83,84,85]. For example, Mader et al. [7] adjusted a THI model for cattle by including wind velocity and solar radiation in the model, and by assessing the level of heat stress comfort through painting scores. The same model was correlated with the milk yield of Holsteins cows in temperate regions [83], which identified a decline in heat stress thresholds. The same tendency was observed by Gorniak et al. [82] in Germany, for Holstein dairy cows but using a different THI model. Gaughan et al. [37] developed a model that considers ambient temperature, humidity, solar radiation, and wind velocity, but instead of one equation, the model has two. The model is called Heat Load Index (HLI), and the two regression equations are set for different black globe temperature ranges (threshold of 25 °C). Further, the effect of different management strategies (e.g., access to shade) and animal-related factors (e.g., genotype and coat color) are considered through specific heat stress thresholds [37]. Other strategies were used to integrate the effect of wind and solar radiation on the index predictions: indirectly, such as using a black globe temperature (in the dairy heat load index (DHLI) [84]), through multiple non-linear equations (indexes CCI and HLI [37,86]) or even considering the weather parameters interaction (equivalent temperature index for cattle (ETIC) [85]).

The thermal state of a heat-stressed animal strongly depends on the intensity and duration of heat-stress conditions, as well as the heat accumulated from previous exposures, both time-related factors, that are often neglected by the broader bioclimatic indexes. Nevertheless, Ji et al. [87] adjusted bioclimatic indexes to simultaneously quantify the intensity and duration of heat stress, considering both short- and long-term effects. Multiple linear regressions were correlated between DMY and the developed indexes considering the animal’s age, body mass and days in milk. Following a different approach, Gaughan et al. [37] developed a model (i.e., accumulated heat load (AHL)) to predict the body heat load balance over time, considering the total hours above a threshold HLI. For HLI values above the threshold, the animal accumulates heat, and the opposite occurs if below the threshold value. The AHL index showed a high correlation with the panting score for different beef cattle genotypes.

Several more advanced indexes have been studied. However, they were developed for different geographic locations, on varied diets, and farm systems. For that reason, numerous studies comparing the performance of bioclimatic indexes under specific conditions [11,88,89], have been drawing distinct conclusions. For example, Van Laer et al. [11] likened six bioclimatic indices and found that the HLI was the best predictor of cow thermal discomfort. The authors evaluated the summer conditions and shade availability for two herds (i.e., Holstein dairy cows and Belgian Blue beef cows) kept on pasture in a temperate area. However, in another study of lactating cows, in this case, housed in a naturally ventilated barn [88], it was concluded that the HLI underestimate the degree of heat stress (originally developed for unshaded animals [37]) while the CCI was indicated as a promising index to estimate heat stress of housed dairy cows. Ji et al. [57] showed a straightforward comparison of bioclimatic indices under several ambient conditions, i.e., temperature (20 to ~35 °C), humidity (~50 to ~86%), wind velocity (0 to ~7 m·s^−1^), and solar radiation (0 to ~1100 W·m^−2^). According to Ji et al. (2020, [57]), it is clear that the use of different bioclimatic indexes can lead to opposing conclusions: from neutral conditions, with one index, to moderate heat stress conditions, with another one, or even odd outcomes. For example, for critical hot-wet conditions, only a maximum level of moderate heat stress was obtained with one of the indexes evaluated (*HLI*_1_ [57]), in opposition to the level of severe heat stress predicted by the remaining indexes. This emphasizes the need to study the index accuracy and possibly adjust the thresholds to both local farm features and cattle traits.

In order to build an algorithm based on the mentioned indexes, the values above which the animal starts feeling uncomfortable need to be defined. However, the threshold values that define if the animal is in mild, moderate, or severe heat stress, are very dependent on the index selected. For instance, as an indicator of a mild level of heat stress, values around 25 (to 30), 65 (to 74), and 70 (to 80) are, respectively, reported in studies based on CCI [86,88], THI [7,34,78], and HLI indexes [11,37]. Additionally, it is unrealistic to assume uniform thresholds for categorizing the level of heat stress of all animals on a farm as the animals have varied biological attributes (age, genotype, and production level), and the animal thermal state is highly dependent on the specific environmental conditions each animal is exposed to. As an example, Ji et al. [90] adjusted the thresholds, by using a machine learning technique to obtain accurate levels of heat stress for individual cows, although uniform thresholds were found adequate to predict the decline of daily milk yield of the herd in a previous study [76].

The effect of animal individual characteristics on heat load threshold has been recently studied [7,34,78,90]. For example, Pinto et al. [78] determined the heat load thresholds of THI based on respiration rate for different postures (standing or laying) of Holstein-Friesian lactating cows. More cow-related factors were considered by Yan et al. [34], namely milk yield, days in milk, and parity, in a study with Chinese Holstein lactating cows. In both studies, the heat load thresholds were significantly affected by the animal physiological and behavior indicators. Though, under a daily farm routine, it is not feasible to monitor the mentioned indicators. For instance, the continuum tracking of an individual cow’s physiological and behavior indicators can be compromised due to technical issues (sensors displacement or total removal), requiring significant expenses on human and equipment resources (e.g., a high number of measurements were reported in studies evaluating painting scores; in Table 1). Under this perspective, it seems reasonable to disregard individual indicators (physiological and behavioral), focusing instead on the measurement of parameters already monitored by the farm system (e.g., milk production) and relying more on relatively inexpensive equipment, such as environmental sensors. Nevertheless, the animal individual response can be considered through mechanistic models as proposed by Berman [91]. The author developed an index based on data simulation of a mechanistic model considering the variety of animal-specific data (e.g., metabolic heat production, skin water loss, coat thickness, tissue and coat insulation). A correlation between respiratory heat loss (HER, [91]), air temperature, velocity and humidity was expressed by two regression equations. Furthermore, a similar mechanistic model was used to evaluate the accuracy of two indexes (THI and CCI) to predict the cow thermal status considering different cooling methods (e.g., fans and sprinklers; [89]). Devoe et al. [89] concluded that the index that accounted for the airspeed (CCI) was the most suitable to predict heat stress level of shaded cows regarding mitigation methods. As already mentioned, another possibility for adjusting the heat stress threshold is the use of machine learning. Ji et al. [90] calculated the thresholds considering the individual variance of body mass, age, and days in milk, as described in the following section.

The predictions of the mentioned bioclimatic indexes are estimations, always associated with a certain degree of uncertainty, that can be minimized by testing and validation of the indexes for the farm conditions, considering the management strategies (e.g., shade and cooling methods), and the animals’ physiological variables. Nowadays, research and development of full extended indexes can benefit from the available technological solutions and techniques to create a virtual environment for testing and refinement, e.g., through machine learning algorithms (Section 3.2) and/or by using mechanistic models (Section 3.3).

**Table 1:** Example of bioclimatic indexes and the corresponding input and output variables.

Study Goal	Population	Inputs	Outputs	Observations	Ref.
Temperature-humidity index (THI) developed for bull calves	Four Ayrshire bull calves (9 months old)	Dry-bulb temperature; Wet-bulb temperature.	Rectal temperature	One equation. For five hours, each animal was kept inside a climate chamber. The trials were repeated 3 times per animal.	[80]
Correlation between milk production and ambient temperature and humidity	56 Holstein cows (with several stages of lactation and production levels ranging from 6 to 70 lb per day)	Dry-bulb temperature; Wet-bulb temperature; Normal production level.	Production level	One equation. A good relationship between a THI index and milk production was obtained. A new equation was proposed considering besides air temperature and humidity also the normal production level.	[92]
Adjusted THI for cattle, considering wind and solar radiation	Three experiments with a varied number of animals (from 72 to 192)	Air temperature; Relative humidity; Wind velocity; Solar radiation.	Panting score	One equation. Required more than 2000 individual panting score assessments derived from ~12 d of observations.	[7]
Linear regression equation to estimate respiration rate of no-shade feedlot cattle	Eight crossbred steers	Dry-bulb temperature; Relative humidity; Wind velocity; Solar radiation.	Respiration rate	Two equations. Responses were studied during eight periods within 4-months. Animals randomly assigned to concrete surfaced pens with shade or no-shade option.	[61]
Respiratory heat loss (HER)	Simulation data	Air temperature; Relative humidity; Wind velocity; Animal-related factors (e.g., coat insulation and thickness)	Respiratory heat loss	Lumped model. Outputs of simulations were used to produce estimates of thresholds of maximal respiratory response as a function of ambient conditions for different cows-related factors.	[91]
Heat load index (HLI)	Feedlot cattle for seven genotypes (more than 10,000 animals)	Air temperature; Relative humidity; Wind velocity; Solar radiation; Animal-related factors (e.g., genotype, coat color, health status).	Panting score	Two equations. Responses were studied for eight summers. Approximately 162 observations were made per animal (3 times per day for 54 days).	[37]
Comprehensive climate index (CCI) for application under a wide range of environmental conditions (hot and cold)	Livestock cattle (number not defined)	Air temperature; Relative humidity; Wind velocity; Solar radiation.	Dry Matter Intake	Multiple non-linear equations. Based on experimental results reported in the literature. Responses were studied for nine summers and six winters. The model performance was compared with wind-chill and heat indexes.	[86]

### 3.2. Machine Learning

Most of the developed bioclimatic indexes assume either linear relationships between environmental factors and the physiological responses, or relationships with a specific form, which are still relatively simple representations of the heat stress on dairy cows. In recent years, several technologies have been developed allowing the collection of a large amount of data about these animals from a generalized use of sensors to automatic milking systems. With the increase in available data, it is also important to use methods that allow a better understanding as well as the treatment of large quantities of data to help a farmer run operations more efficiently. These methods leverage data to go beyond linear relationships and other simple models. Among them, are the machine learning (ML) tools [93].

#### 3.2.1. Fundamentals of Machine Learning (ML)

Machine learning is a powerful concept, part of Artificial Intelligence, that enables systems to learn complex non-linear relationships in data without being explicitly programmed to do so [94,95]. By learning by themselves, machine learning systems also minimize bias regarding the expected relationships, such as between environmental factors and physiological responses [96]. Machine learning has been used in different areas from petroleum reservoir characterization [97] to predictions in the stock market [98]. ML has also been applied to assess dairy cow behavior as well as milk production [99,100], and ultimately the effect of heat stress on the cow’s body response [101].

Machine learning has three main categories: supervised, unsupervised, and reinforcement learning. Most studies dedicated to predicting dairy cow behavior are supported by supervised learning methods, thus, a brief description of these methods is provided.

Supervised learning means that the model is fed with a set of inputs for which the outputs are known. The program already knows the result for an initial set of conditions. The algorithm will then learn the necessary steps that allow it to go from the inputs to the outputs. This type of machine learning is one of the most prevalent and it tackles different scenarios: regression (outputs are real numbers) or classification (the outputs are categorical; [102]). Supervised learning algorithms include linear regression, logistic regression [103], naïve Bayes, Support-Vector machines [104], decision trees [105], and Artificial Neural Networks (ANN) as well as several variations of these and other entirely separate algorithms altogether. These algorithms can also be combined with different methods to improve their functionality.

**Figure 1:**
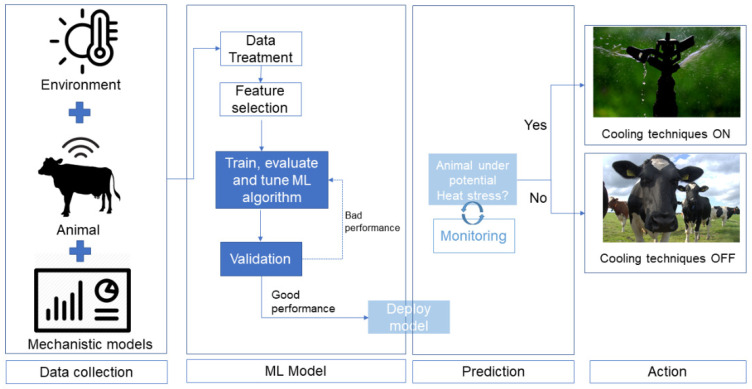
General tasks associated with the implementation of ML techniques to predict the herd thermal state.

These systems are data-driven so the right quality/quantity of data points is essential to have a reliable tool to attend to the problem. As such, the first requirement to implement this type of method is to have an adequate dataset. A part of this data (60% to 80%) will be used to train the algorithm. Once a model is trained, the ability of the model to predict new results is tested against new values that were not part of the training data set. The approach can be iterated upon and changed until the model shows a good prediction performance. At this point, the model can be used for new inputs for which the outputs are not known. Figure 1 shows a schematic representation of how the ML can be used to predict a heat stress event and help the farmer in choosing when to turn on or off the cooling techniques.

Building a highly accurate prediction model comes with multiple challenges such as which data and features to use, which algorithm to choose, how to deal with large amounts of data, as well as how to guarantee the performance of the model in a real-world context.

#### 3.2.2. Prediction of Heat-Stressed Cows

As previously mentioned, the use of ML in the domain of cattle has already been reported in the literature. This chapter focuses on the use of these technologies to predict events or possible indicators of heat stress on cattle. A robust tool able to predict the conditions in which the animal will face heat stress is the desired output. This will provide a non-invasive method to prevent the animal from being exposed to extreme conditions while allowing more efficient management of the resources (water/energy) to cool down the animal.

In several studies, ML is used to predict physiological responses (outputs), from the environmental variables as well as other easy-to-measure inputs. Table 2 shows a summary of some machine learning studies and the corresponding input and output variables.

One of the first studies aiming to evaluate heat stress on heifers was presented by Brown-Brandl et al. [106]. This study considered different techniques to predict the physiological response (in the form of respiration rate) to evaluate if the animal was under heat stress. As inputs, several environmental parameters were considered (dry bulb temperature, dew point temperature, solar radiation, and wind speed) as well as the breed of the animal (average temperatures of hair coat surface in the afternoon; animal color). The authors concluded that different breeds are affected differently by environmental conditions, and as such, taking into account the breed is necessary to predict the effect of the different factors on thermal stress. In terms of models, it is shown that ANN is slightly better at predicting respiration rate than regressions. However, if more information is desired, other than simply the value of the physiological response, fuzzy inference systems (data-dependent) may be more useful. The authors also pointed out that even though these techniques are promising, the available data was not yet enough to lead to accurate predictions. Furthermore, they suggested that a factor accounting for heat accumulation could be a method for improving these models.

The work of Hernandez-Julio [101] also uses different ML tools to predict the physiological response of dairy cows (rectal temperature and respiratory rate) based on dry-bulb air temperature and relative humidity as inputs. Two ANNs are proposed to obtain the physiological response of the animal (one for each output), based on both experimental data and data from the literature. The latter avoids carrying out further experiments, hence minimizing the costs related to equipment and human resources.

Another example of using ML to predict the physiological response of the animal is the work presented by Gorczyca and Gebremedhina [96]. This study evaluated how the environmental factors (air temperature, relative humidity, solar radiation, and wind speed) influenced physiological responses (respiration rate, skin temperature, and vaginal temperature) and it also tried to rank the environmental factors according to their “contribution” on heat stress events. This study, once again, showed the usefulness of ML algorithms in predicting the physiological response of the animal. Besides, it indicated air temperature as the factor that had the highest impact on the physiological response, while wind speed had the lowest.

Sousa [107] performed a study to predict the rectal temperature (an indicator of heat stress) of feedlot cattle using the head surface temperature of the animal (IRT), dry bulb temperature, and wet bulb temperature. The predicted and measured rectal temperatures were classified on levels of thermal stress and compared with the classification based on traditional THI (temperature–humidity index). An ANN and a linear regression model were compared. The ANN led to models that are better at predicting the rectal temperature than standard correlations, in addition to allowing individual assessments.

Hempel [8] showed a different approach to the heat stress prediction problem. The goal was to infer the conditions inside the barn at any moment just by assessing the environmental conditions near it. Using the conditions inside the barn, the authors used empirical models to evaluate the comfort and discomfort of the animals—THI and ETIC (equivalent temperature index for cattle). Economic and environmental impacts were also estimated based on the heat stress events (heat stress risk).

**Table 2:** Example of machine learning studies and the corresponding input and output variables.

Study Goal	Population	Inputs	Outputs	Algorithms	Ref.
Evaluate the heat stress of cattle	128 heifers	Environmental data: dry bulb temperature, dew point temperature, solar radiation, wind speed. Animal related parameter: temperature of the hair coat color.	Respiration rate.	Regression models; Fuzzy inference systems; ANN.	[106]
Predict the physiological response of dairy cows	Holstein dairy cows (experimental + literature data)	Environmental data: dry-bulb air temperature, relative humidity.	Rectal temperature; respiratory rate.	Regression; ANN; Neurofuzzy networks.	[101]
Effect of the environmental factors on physiological responses	19 dairy cows	Environmental data: air temperature; relative humidity, solar radiation, and wind speed.	Respiration rate; Skin temperature; Vaginal temperature.	Penalized linear regression; random forests; Gradient boosted machines; ANN.	[96]
Predicting the heat stress for feedlot cattle	26 Nellore steers	Environmental data: dry and wet bulb temperature. Physiological parameter: temperature of head surface.	Rectal temperature.	Correlations; ANN.	[107]
Evaluate the heat stress in naturally ventilated barns for dairy cows		Outdoor conditions: temperature, relative humidity, zonal and meridional wind, sea level pressure, and global radiation;	Conditions inside the husbandries: temperature, relative humidity, and wind components.	Linear regression with and without regularization; random forest; ANN; Support-vector models.	[8]
Definition of dynamic thresholds for heat stress alerts	126 cows	Environmental data: minimum and mean ambient temperature. Body mass, days in milk, daily milk yields, and milk temperature.	Heat stress thresholds were redefined for the herd taking into consideration the daily milk yield and milk temperature.	Decision tree	[90]
Evaluate the milk yield under different thermal conditions	Holstein-Friesian cows	Air temperature around cowsheds.	Milk yield	ANN	[99]
Best cow treatment to improve the milk yield	dairy cows in Indonesia	Environmental data: temperature, wind speed, and relative humidity. Physiological parameters: heart rate, body temperature.	Milk yield	ANN	[108]
Prediction of the milk yield	91 dairy cows	Barn environmental data: relative humidity and temperature. Days in milk of the cow.	Daily milk yield	Random forest	[100]

Ji et al. [90] also used ML techniques to define dynamic thresholds for heat stress alerts with auto-calibration. Data to be used was collected from a robotic farm: body mass, days in milk, daily milk yields, and milk temperature, as well as ambient temperature (minimum and mean value). Heat stress thresholds were redefined for the herd taking into consideration the daily milk yield as well as the milk temperature. Then, at an individual scale, a decision tree (machine learning model) was used to artificially define new thresholds where specific groups were summarized as classification factors (age, body mass, and days in milk). Taking the minimum (representing night-time cooling conditions) and the mean temperature (daily thermal conditions), these models were able to predict new heat stress thresholds. The authors also stated that while a large quantity of data was used, it all came from a single farm. Therefore, data from other sources are desirable to generalize these conclusions.

Some studies do not deal with heat stress directly but report other parameters (like milk quality) that can be an indicator that the cow is under thermal stress. One example is the work of Bonieck et al. [99]. In this research, the authors used air temperature around cowsheds (maximum temperature of the day) to build an ANN capable of predicting milk yield under different thermal conditions.

Sugiono et al. [108] also used ML to develop a model for the selection of the best cow management to improve milk yield. Once again, the main goal was not to evaluate the heat stress, but it did take into consideration environmental data (temperature, wind speed, and relative humidity) and physiological aspects (heart rate and body temperature) to correlate with milk yield. A sensitive analysis was also performed, and it pointed to relative humidity, heart rate, environment temperature, and cow body temperature as the factors that had a higher effect on milk production. The authors still recommended the improvement of this model by adding more points, testing different learning algorithms, and including other factors such as age and weight, among others.

Bovo et al. [100] used a random forest algorithm to evaluate the daily milk yield of a single cow. The authors used data from dairy cows collected by automatic milking systems (AMSs) as well as the environmental conditions of the barn (relative humidity and temperature) that were incorporated in the form of THI. This algorithm considered the THI on the test day and on the previous five days. It was tested for the data set available and the authors reported that it could predict the daily milk yield with a relative error of just 18%.

Overall, these studies showed that machine learning can be very useful to help predict/monitor heat stress to which the animals are exposed. However, as some of these works have pointed out, much remains to be done. More data are necessary, as well as a better understanding of the critical factors (like accounting for heat accumulation) to predict heat stress. Once reliable tools to predict heat stress are available, it will be possible to trigger the cooling systems only when necessary and minimize the use of invasive techniques to obtain information from the animals. As mentioned by Gorczyca [96], even though this is a powerful set of tools it does not mean that these algorithms/models will be consistently the best option to predict heat stress. This is in part because computational and large amounts of data resources are necessary to train and optimize these methods but may not always be available. As an option, alternative mathematical models (mechanistic models) can be used to generate the necessary numerical data to feed the machine learning models. Different types of input variables can be assumed from environmental and animal data to numerical results obtained through mechanistic models.

### 3.3. Mechanistic Models

**Figure 2:**
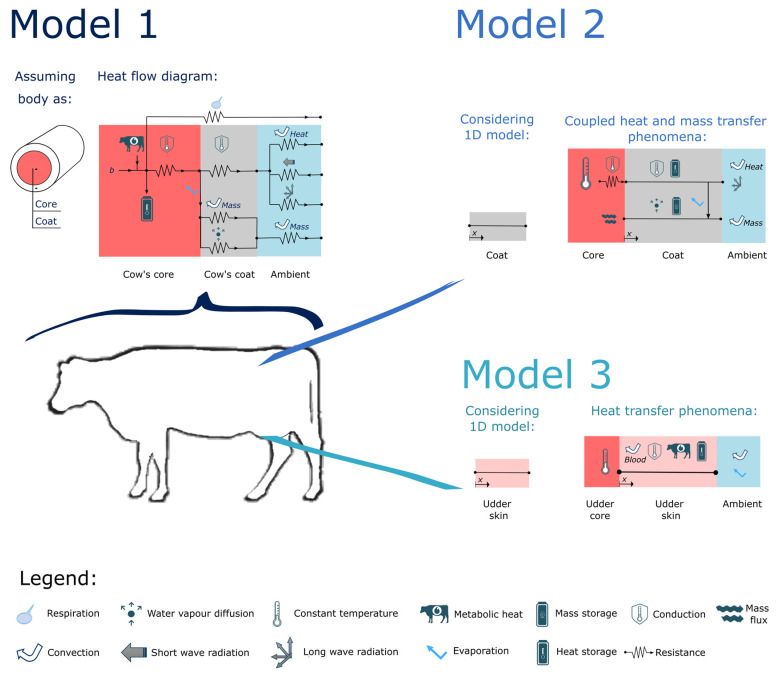
Representation of three independent mechanistic models developed for different zones of the cow: entire body (model 1, [114]), coat (model 2, [110]), and udder (model 3, [115]).

A mechanistic model can be defined as a mathematical model that correlates physical phenomena in a deterministic way. The mathematical models used to predict animals’ thermal responses are few and extremely restricted to the considered scenario (e.g., neglecting body region characteristics [109,110,111]). Even fewer models consider cattle thermoregulation mechanisms (e.g., panting, sweating; [112,113,114]). Nevertheless, the latter model [114] has the potential to simulate the dynamic thermal balance of the cow. As the main assumption, the animal body is assumed as one horizontal cylinder with closed ends, and the model has three layers: core, skin, and coat (model 1, Figure 2). As a lumped element model, the calculated loads at every time step assume a quasi-steady state. Furthermore, the model allows the study of a wide combination of environmental conditions (temperature, humidity, air velocity, and solar radiation). However, only one asymmetrical environment condition option is considered (i.e., exposed area to shadow or direct sunlight) and, for that reason, it is not possible to analyze the effect of non-uniform distribution of environmental conditions on cow thermal state (e.g., it is not possible to simulate the heat transfer between the body region of a laying cow and the ground). To approximate realistic scenarios, it is necessary to determine proper boundary conditions by body region. Additionally, another relevant requirement is the reference values used to characterize the neutral conditions (e.g., temperature by body layers) according to the cow’s physical conditions (e.g., age, days in milk).

The McGovern and Bruce model [114] could not be used to predict the cow thermal balance at low temperatures since cold-induced thermogenesis is neglected. This simplification can put at stake the accuracy of the analyses of cooling technologies’ performance, or, in some cases, the prediction of cow cooling during the night. Furthermore, proper cutaneous and respiratory evaporation rates must be considered [91,116], since the original model set some correlations (e.g., tidal volume of the respiratory system) and animal parameters (e.g., tissue thermal resistance) based on the available literature on different breeds [114], and, consequently, only the qualitative behavior of the numerical results were assessed. Berman [91] adjusted seven animal parameters in the model, specifically for the Holstein cow breed. The author concluded that this modification increased the accuracy of total skin and respiratory heat loss predictions. However, the described validation assumptions do not allow us to infer the accuracy of the cow thermal state predictions during highly transient ambient conditions. Nevertheless, these mechanistic models were used to develop a bioclimatic index considering animal-related factors [91] and to create contour maps with optimal cooling system recommendations throughout the United States [89].

At the surface of the cow body, the sensible and latent heat losses are generally ruled by convection (heat and mass) and radiation phenomena. For that reason, and to reduce the effect of thermal stress on dairy cattle, the main cooling technologies are shade structures to diminish incoming radiation, forced convection to increase heat/mass removal or water to remove heat by evaporation. To predict the evaporative and convective heat loss from a cow body, Gebremedhin and Wu [117] developed a one-dimensional model, considering the heat and mass transfer model along with the hair coat of a cow, in steady-state conditions. Several parameters can be studied as different levels of wetness, air velocity, ambient temperature, relative humidity, and hair properties. As a practical application, the model can provide insights into the effectiveness of evaporative cooling such as, for example, ventilated spaces [118] or water sprays coupled with fans [110]. Gebremedhin and Wu [118] studied the (sensible and latent) heat loss from cattle in a ventilated space occupied by 10 cows. It should be noted that the fluid flow around each cow is highly dependent on the air inlet velocity and the geometric features of the surrounding obstacles (i.e., chamber dimensions and other cows). Therefore, numerical simulations were conducted to characterize the fluid flow around the cattle using Computation Fluid Dynamics software. Therefore, the numerical results of fluid flow were used as inputs of the coupled model of heat and mass transfer through cow fur. The total heat loss from the cows changed from 215 to 710 W due to the different characteristics of the flow field surrounding the animals. A similar one-dimensional model was used by Chen et al. [110] to study the transient behavior of wetted fur (model 2, Figure 2). The authors estimated the drying time and heat removal as a function of ambient conditions (air temperature, air speed, humidity, and mean radiant temperature). Furthermore, the numerical results were used to develop a correlation for a control algorithm of a sprinkler–fan system. The equipment frequency was related to cooling load thresholds obtained for outdoor conditions. Moreover, the use of the control algorithm was estimated to reduce electricity and water consumption by 25% and 50%, respectively. This highlights the potential ability of mechanistic models to improve the performance of cooling systems while contributing to a more sustainable dairy industry.

An often-used mechanistic model to simulate heat transfer in human tissues is the Pennes’ bioheat model [119,120], which was the same used by Gebremedhin and Wu [115] to predict the udder heat loss in cows under different conditions of ambient temperature. At the udder skin (model 3, Figure 2), the heat was transferred to the environment by convection and eliminated by evaporation. At the core of the udder, a fixed temperature was assumed. The model quantifies the total heat loss, which can be useful to estimate the performance of cooling technologies that promote convection and/or evaporation [115]. Furthermore, this model can be used to study the thermal behavior of different body regions under different scenarios of air velocity and temperature.

The potential of artificial cooling methods to alleviate the heat stress of dairy cattle has been proven. However, the economic benefits of a particular cooling method depend on farm features and geographical location. Thus, the benefits/limitations should be assessed prior to the installation of any equipment [74,89]. For example, Herzog et al. [121] concluded that the advantage of using basket fans for heat abatement is slightly higher than the environmental costs associated with fan production and operation, highlighting the necessity of studies based on primary data regarding the effectiveness of fan cooling to improve cow productivity. Mechanistic models can also be used as a support tool to analyze the environmental impact of the equipment’s implementation. Based on numerical data, Devoe et al. [89] obtained multiple maps of the U.S. that help producers determine which cooling strategy is the most economical in their region.

The complexity of the mechanistic models (several input variables and equations) makes them difficult to be used by an ordinary end-user without a background in transfer phenomena [58]. However, and to overcome this problem, the mechanistic models can be integrated as predictive algorithms for controlling systems, running in the background, and being automatically fed by input variables measured through emergent monitoring technologies (e.g., sensors/wireless/cloud-based).

Table 3 shows a summary of some studies that used mechanistic models and the corresponding input and output variables. 

## 4. Future Considerations

As a predictive algorithm, the mechanistic models have more degrees of freedom and flexibility to be easily adapted to ever-changing situations (e.g., days in milk of the cows) or to assess several types of cooling technologies (e.g., basket fans, sprinklers) than the bioclimatic indexes and machine learning algorithms.

The next step in the development of more robust mechanistic models is the incremental improvement of the thermal model complexity by considering a virtual cow with different body segments, following the approach often used in human thermoregulation models: fitting the physiological dimensions to a suitable geometry [115]. Furthermore, more layers can be considered in each body segment (i.e., core, muscle, fat, and skin), and a central core to which the heat of each body layer is transferred by blood flow [122]. Therefore, it is necessary to determine the distribution of the heat generated along body segments and layers due to different mechanisms (e.g., heat generated by external work occurs only in the muscle layer). Another relevant requirement is the definition of reference values of parameters/properties obtained in neutral conditions, such as the temperature of body layers, or local heat produced (metabolic heat), according to the cow’s physical conditions (e.g., age, days in milk).

The simulation of a countless number of scenarios with precise outcomes entails the definition of proper boundary conditions of heat and mass transfer phenomena. For instance, the fluid flow around the animal body is dependent on the animal posture (laying or standing), the location (inside/outside the barn), and the surrounding obstacles (trees or other cows), and it will be significantly different between body regions (e.g., due to geometry considerations, and hair coat density). This affects the net of heat and mass exchange between the cow skin and the environment and further studies should be carried out to assess realistic heat and mass transfer coefficients. Radiation is another relevant heat transfer phenomenon, which is significantly constrained by the surrounding surfaces’ thermal state (e.g., region–region, body–environment, and cow–cow body exchange). Representative boundary conditions of radiation should be studied, consisting, e.g., in the determination of suitable radiative coefficients distribution along the different body regions.

Additionally, the results of these mechanistic models combined with data collected from real case scenarios can be used as inputs to develop new and more robust machine learning models (as proposed in Figure 1). Machine learning can be a useful tool to predict heat stress conditions, but it requires significant quantities of data to make reliable predictions about any phenomena. Mechanistic models, as well as the generalized use of sensors, can be useful to obtain a great volume of important information on heat stress conditions, which may help to overcome the actual problems associated with the use of machine learning models. Besides, the continuous monitoring of several features associated with animal well-being and productivity (in a robotic farm, for example), may also be useful to monitor and improve the performance of these tools.

## 5. Conclusions

Under certain environmental conditions, only cooling systems are efficient to mitigate dairy cattle heat stress. The integration of predictive algorithms in the operation of these systems is a possible method of efficiently regulating environmental conditions while reducing the associated energy and water consumption. Three potential approaches to be used as predictive models were analyzed in this review.

Bioclimatic indexes have been studied for several years, and they were developed for different geographic locations, on varying diets and farm systems. From a practical perspective, the selection of a suitable bioclimate index for a farm implies carrying out a local study, which is time-consuming and can also be expensive. Furthermore, the typical bioclimatic indexes are based on average daily measurements, presenting direct relationships between the environmental factors. Additionally, they consider the general physiological conditions of the herd, rather than the individual or herd-specific characteristics. In a complementary way, machine learning and mechanistic models are two methods capable of predicting individual and herd responses, assuming more complex relationships between factors.

The application of machine learning to predict the cow’s discomfort level is a recent trend, with several studies using ML to predict physiological responses. The application of these models can provide a non-invasive tool that predicts the conditions that lead to heat stress in animals, providing information on when and for how long to use cooling types of equipment. Nevertheless, one must consider important limitations such as the apparent inability to understand the fundamental aspects of heat stress in dairy cattle and the need for large amounts of data, with more efficient and comprehensive models implying the acquisition of data from more sources. The use of an ML model in a specific farm implies, at least, the validation of the results and, in the worst-case scenario, carrying out a new set of experiments to fine-tune the model. As an alternative, mathematical models (mechanistic models) can be used to generate numerical data to feed machine learning models.

Mechanistic models are a less popular approach than bioclimatic indexes and machine learning but they are the most transversal ones. Their capacity to simulate the individual cow’s thermal state, exposed to countless dynamic ambient conditions, offers an advanced tool for research, development of new bioclimatic indexes, and training of machine learning algorithms. Currently, there are several mechanistic models that allow us to study at different scales (e.g., considering only the animal hair coat or the entire body of the cow), with the potential to evaluate the efficiency of cooling technologies or management strategies (e.g., early access to pasture).

We believe that the integration of suitable predictive models will be a step forward for dairy industry productivity, animal welfare, and the research and development of more efficient technologies for the sustainable use of water and energy.

## Figures and Tables

**Table 3:** Example of mechanistic model studies and the corresponding input and output variables.

Model Description	Main Mathematical Assumptions	Inputs	Outputs	Observations	Ref.
Thermal balance for cattle in hot conditions.	Three node model: core, skin, and coat. Main transfer phenomena at the cow surface (skin + hair): heat transfer by convection and radiation, and mass transfer by convection. Main thermoregulation mechanisms: panting and sweating.	Environmental conditions: temperature, humidity, air velocity, solar radiation. Animal-related parameters: e.g., weight, metabolic heat, body-specific heat, coat reflection coefficient and thickness.	Core, skin, and coat temperature. Sensible and heat loss from respiration. Stored heat. Latent heat loss from the skin.	Only qualitative behavior of the numerical results was assessed.	[114]
Thermal balance for Holstein cows in hot conditions.	Based on [118]. Additionally, the author adjusted several animal-related parameters.	Based on [118].	Based on [118].	Improvement of the accuracy of total skin and respiratory heat loss prediction.	[91]
Thermal balance of livestock.	Three node model: core, skin, and coat. Main transfer phenomena at the cow surface: convection, evaporation, radiation, and solar radiation gain (for animals outdoors). Furthermore, considers the rain effect. Physiological responses: vasomotor action, sweating and panting.	Environmental conditions: temperature, humidity, air velocity, precipitation, direct and diffuse solar radiation. Animal-related parameters: e.g., tissues thermal resistance.	Skin and coat temperature Sensible and latent heat loss.	Simplification of the physical and physiological mechanisms to simulate long data sets for climate change impact analysis.	[109]
Simulation of udder heat loss.	One dimensional approach (heat transfer through skin; from core to ambient). Main phenomena at the skin surface: convection and sweat evaporation. Main phenomena through the skin: conduction, convection (heating by infused blood flow), and metabolic heat production.	Environmental conditions: air temperature and velocity. Animal-related factors: e.g., tissue density and specific heat, metabolic heat production.	Udder skin temperature. Evaporative heat loss from the udder skin. Convective heat loss from the udder skin.	The approach can be used to study the heat loss of other body zones and the performance of cooling technologies (e.g., fans).	[115]
Heat and mass transfer model to estimate drying time of a wetted fur.	One dimensional approach. Simulation domain: hair coat. Main phenomena: heat conduction, diffusion of water vapor, and evaporation.	Environmental conditions: air temperature, humidity and velocity. Animal-related factors: e.g., tissue thermal resistance, fur thermal conductivity, coat thickness.	Skin temperature. Total heat flux at the skin and coat surfaces. Water mass fraction.	It can be used to study the efficiency of water sprays coupled with fan-induced air flow.	[110]
Heat loss from cattle randomly distributed along a ventilated barn.	Domain: ventilated space occupied by 10 cows. Fluid flow fields characterized through 3-dimensional simulation. Cows thermal balance calculated through a coupled heat and mass transfer model (based on [114,121]).	Environmental conditions: air temperature, humidity, and air velocity. Animal-related factors: e.g., tissue thermal resistance, fur thermal conductivity, coat thickness, and animal position inside the barn.	Fluid field around each cow. Skin temperature. Total heat loss for each cow convective and radiant heat losses, sensible and latent heat components).	The approach can be used to obtain realistic convective heat and mass transfer coefficients, assuming different cows’ dimensions and spatial distribution.	[118]

## Data Availability

Not applicable.
